# High-Riding Vertebral Artery in Cervical Spine Surgery: A Review of Preoperative Identification and Surgical Implications

**DOI:** 10.7759/cureus.107592

**Published:** 2026-04-23

**Authors:** Alexander Kucherina, Paul G Mastrokostas, Themistocles S Protopsaltis, Charla R Fischer

**Affiliations:** 1 Orthopaedic Surgery, New York University (NYU) Langone Health, New York, USA; 2 Orthopaedic Surgery, State University of New York (SUNY) Downstate Health Sciences University, Brooklyn, USA; 3 Orthopaedic Surgery, New York University (NYU) Grossman School of Medicine, New York, USA; 4 Orthopaedics, New York University (NYU) Langone Orthopedic Hospital, New York, USA

**Keywords:** atlantoaxial fixation, c2 pedicle screw, cervical spine surgery, ct angiography, high-riding vertebral artery, laminar screw, pars screw, spinal instrumentation, vertebral artery injury

## Abstract

High-riding vertebral artery (HRVA) is an important anatomical variant of the vertebral artery that poses significant challenges during cervical spine surgery, particularly at the C1-C2 level, where it reduces the safe corridor for pedicle screw placement and increases the risk of arterial injury.

This narrative review summarizes current evidence regarding the definition, prevalence, imaging identification, and surgical implications of HRVA, along with risk mitigation strategies. Radiographically, HRVA is most commonly defined by reduced C2 isthmus height (C2IsH) (≤5 mm) and/or internal height (C2InH) (≤2 mm) on computed tomography (CT) scans. Reported prevalence varies widely across populations, typically ranging from 10% to 25%, with higher rates observed in selected patient cohorts. The presence of HRVA necessitates careful perioperative planning, including comprehensive imaging and modification of surgical techniques, such as the use of alternative fixation strategies, including pars screws, laminar screws, navigation-assisted instrumentation, and artery mobilization. Advances in CT angiography (CTA), alternative fixation strategies, surgical navigation, and emerging predictive models may further improve risk stratification and operative safety. Recognition of HRVA and tailored surgical planning are essential to minimize the risk of vertebral artery injury (VAI) and to optimize patient outcomes in cervical spine surgery and instrumentation.

## Introduction and background

Cervical spine surgery is frequently performed to treat a variety of cervical pathologies, including degenerative disease, trauma, tumors, and congenital abnormalities [1,2]. Surgical procedures such as anterior cervical discectomy and fusion, anterior cervical corpectomy, posterior cervical fusion, and atlantoaxial instrumentation have become standard techniques for restoring spinal stability and decompressing the cervical spine [1]. With advances in instrumentation and imaging technology, surgical outcomes have improved substantially; however, these procedures remain associated with potentially serious complications, including vascular injury [1,2].

Among the most feared vascular-related complications of cervical spine surgery and instrumentation is injury to the vertebral artery [1-3]. The vertebral arteries supply the posterior circulation of the brain and course in close proximity to critical surgical regions in the cervical spine [1-3]. Vertebral artery injury (VAI) on its own is relatively uncommon, with reported incidence rates ranging from approximately 0.1% to 1.4% [3]. When the vertebral artery is injured, this may result in catastrophic consequences, including massive hemorrhage, stroke, pseudoaneurysm formation, or even death [1-3]. Therefore, a robust understanding of vertebral artery anatomy and its potential variations is essential for spine surgeons. 

One anatomical variant that has garnered increasing attention is the high-riding vertebral artery (HRVA). HRVA refers to an anatomical configuration in which the vertebral artery courses more medially and superiorly within the C2 vertebra, resulting in reduced pedicle height and narrowing of the safe zone for instrumentation [3]. This variation is particularly relevant during posterior cervical instrumentation, especially in procedures involving C1-C2 fixation [1-4]. Placement of C2 pedicle screws in the presence of HRVA significantly increases the risk of VAI due to the limited corridor available for safe screw placement [4].

The prevalence of HRVA varies across populations and imaging studies, with reported rates ranging from approximately 10% to 25%. Identification of this anatomical variant is therefore critical during preoperative planning [5,6]. Advances in imaging modalities, such as computed tomography (CT) and CT angiography (CTA), have improved the ability of surgeons to identify vertebral artery anomalies prior to surgery [3-6]. Recognition of the presence of HRVA may influence surgical decision-making and consideration of alternative fixation techniques, including C2 pars screws, C2 laminar screws, or navigation-assisted instrumentation [5,6].

Despite the clinical importance of HRVA, the literature describing its prevalence, radiographic identification, and surgical implications remains heterogeneous and not all-encompassing in terms of describing initial evaluation, surgical planning, and risk management strategies [1-6]. Definitions of HRVA also vary greatly among studies, and there is no universally accepted radiographic criterion [5,6].

The purpose of this narrative review is therefore to summarize the current evidence regarding HRVA in cervical spine surgery. Specifically, this review aims to (1) describe the relevant vertebral artery anatomy and definitions of HRVA, (2) examine reported prevalence and imaging identification methods, and (3) discuss the surgical implications of HRVA and strategies for mitigating the risk of VAI during cervical spine instrumentation. 

## Review

Vertebral artery anatomy

The vertebral arteries are critical components of the posterior cerebral circulation and are highly relevant in cervical spine surgery due to their close proximity to commonly used surgical corridors [7]. Each vertebral artery originates from the subclavian artery and ascends through the cervical spine before entering the cranial cavity, ultimately contributing to the formation of the basilar artery [7]. Anatomically, the vertebral artery is divided into four segments: the V1 segment (origin to C6 transverse foramen), the V2 segment (C6-C2 transverse foramina), the V3 segment (C2 exit to dural entry at the foramen magnum), and the intracranial V4 segment [7]. This segmentation is clinically important, as surgical risk varies depending on the location of the artery along its course with respect to the nearest cervical vertebra [8]. For example, anterior cervical decompression carries a 0.18% VAI risk, primarily from drilling and instrumentation [8,9]. Meanwhile, C1-C2 posterior fusion has the highest risk at 1.35%, with C1 lateral mass and C2 pedicle screws accounting for most injuries, whereas C3-C7 posterior fusion has a much lower risk at 0.20% [8,9].

Of particular surgical relevance is the V2 segment, which ascends through the transverse foramina from C6 to C2, lying in close proximity to the cervical pedicles and lateral masses [7,10]. At C2, the artery occupies the transverse foramen adjacent to the C2 pedicle and pars interarticularis, which are key bony corridors used for screw fixation during atlantoaxial instrumentation [7,9-13].

After exiting the C2 transverse foramen, the artery enters the V3 segment. Here, it follows a complex, tortuous course: it first travels superiorly and laterally to pass through the C1 transverse foramen, then curves medially along the superior surface of the C1 posterior arch within the vertebral artery groove (sulcus arteriosus). From this groove, it pierces the atlanto-occipital membrane and dura before entering the foramen magnum and the cranial cavity [9-13].

This V2-V3 transition zone is particularly vulnerable during posterior cervical exposure and instrumentation. Several features account for this risk: (1) the artery loses the bony protection of the transverse foramina as it courses over the C1 arch, (2) it lies superficial and lateral within the surgical field during high cervical dissection, and (3) its trajectory shows substantial anatomical variation, including fenestrations, persistent fetal origins, and a high-riding course over the C2 pars that can preclude safe screw placement in up to 20% of patients [7,14].

The passage of the vertebral artery through C2 demonstrates substantial anatomical variability. In some cases, the artery forms a “vertebral artery cave,” a concavity within the C2 pedicle or vertebral body, thought to result from chronic arterial pulsations [10-13]. This morphological variation further reduces the available osseous corridor for instrumentation and may compromise pedicle integrity [10-13]. Even in normal anatomy, the relationship between the vertebral artery and the C2 pedicle creates a narrow safe zone for screw placement [10-13]. Variations such as medialization or superior displacement of the artery within the transverse foramen can significantly reduce pedicle height and increase the risk of VAI during instrumentation [10-12].

Anatomical variations of the vertebral artery are common and include asymmetry in vessel dominance, anomalous entry into the transverse foramina (e.g., entry at C5 or C7), duplication, and variations in the course of the artery around the C1-C2 complex [10-18]. For example, medial loop formation is an anatomic variation in which the vertebral artery extends medially inside the uncovertebral joint and often goes undetected on imaging [17]. However, among these variations, the HRVA is of particular clinical importance. HRVA is characterized by a more medial and superior course of the artery within the C2 vertebra, resulting in reduced pedicle height and narrowing of the safe corridor for instrumentation [12,15]. In such cases, the artery may course above or encroach upon the C2 pedicle, markedly increasing the risk of injury during pedicle screw placement [4-6,15,16]. Additionally, small branches arising from the V2 segment at this level contribute to spinal cord perfusion through anastomoses with segmental and radicular arteries, making vascular injury in this region potentially catastrophic [7-10]. Abnormal vertebral artery entry levels occur in 5%-8% of HRVA cases, with entry at C5 (4.7%), C4 (2.5%), or C7 (0.9%) instead of the typical C6 level [18].

Given this variability, accurate preoperative assessment of vertebral artery anatomy is essential. CTA allows detailed visualization of both osseous and vascular structures and is critical for identifying HRVA and other anatomical variants before C1-C2 instrumentation [19-21]. Careful evaluation of these relationships enables surgeons to plan appropriate screw trajectories and select alternative fixation strategies when necessary, thereby minimizing the risk of vascular complications [19-21]. 

Definition and radiographic identification of HRVA

HRVA is an anatomical variant characterized by a more superior and medial course of the vertebral artery at the level of the C2 vertebra, resulting in a reduced osseous corridor for safe pedicle screw placement [5,6,11,13-17]. HRVA is most commonly identified using CT or CTA, which allows precise assessment of the relationship between the vertebral artery and surrounding osseous structures, including the C2 pedicle and pars interarticularis [12,15,16,19-23]. CTA, in particular, provides additional information regarding vascular dominance and anomalous arterial course, which may further influence surgical planning [19].

**Figure 1:**
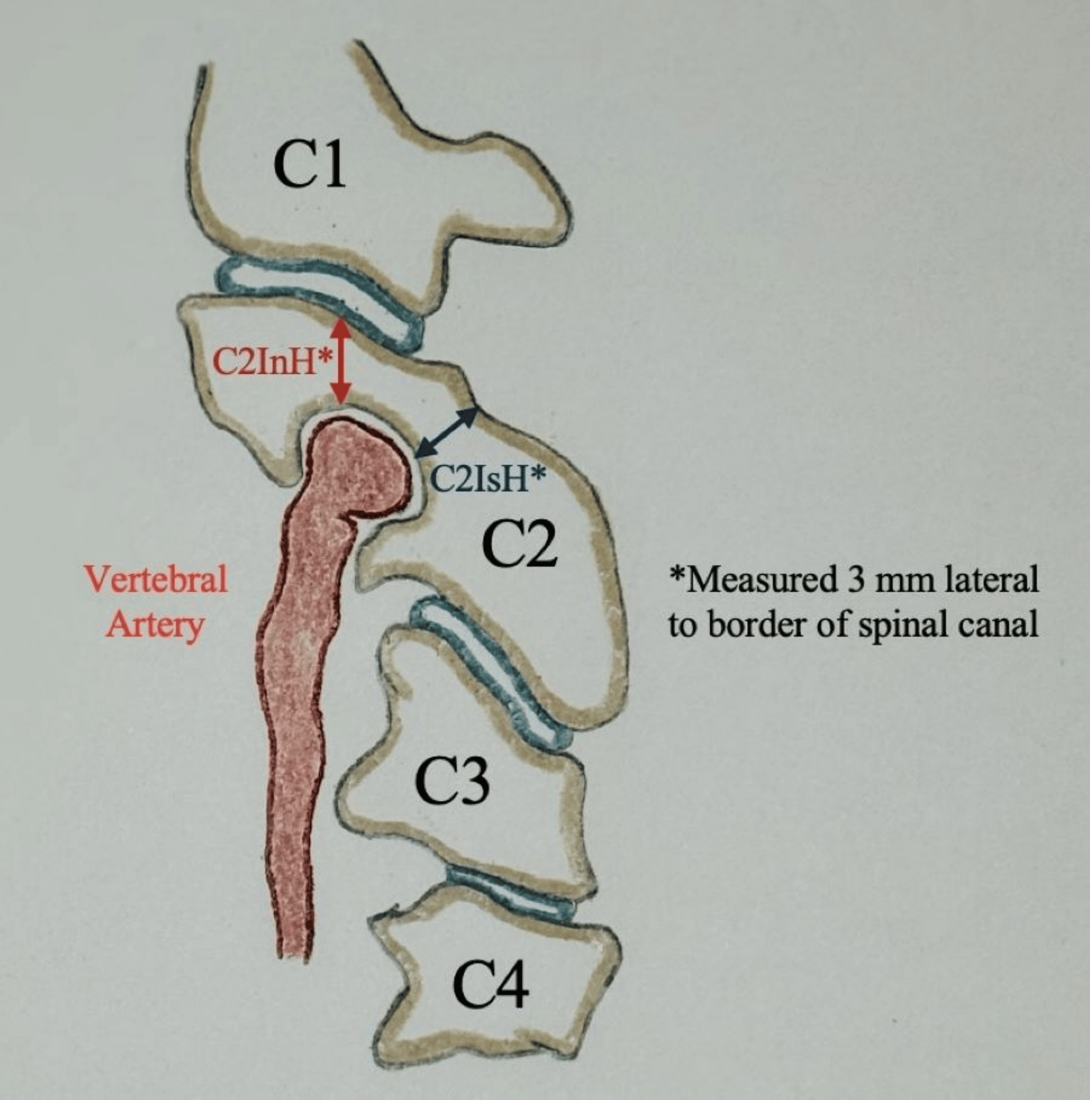
Radiographic measurements used to define high-riding vertebral artery (HRVA). Schematic of the C2 vertebra and vertebral artery demonstrating the measurements used to identify HRVA. The C2 isthmus height (C2IsH) and C2 internal height (C2InH) are measured approximately 3 mm lateral to the lateral border of the spinal canal. HRVA is typically defined as C2IsH ≤5 mm and/or C2InH ≤2 mm. This image is an original, author-created schematic using PowerPoint (Microsoft® Corp., Redmond, WA, USA).

Radiographically, HRVA is most commonly defined using measurements obtained from sagittal CT imaging [24-26]. Figure 1 illustrates the radiographic parameters used to identify HRVA. Classic criteria define HRVA as a C2 isthmus height (C2IsH) ≤5 mm and/or C2 internal height (C2InH) ≤2 mm, measured on sagittal images approximately 3 mm lateral to the lateral border of the spinal canal [5,6,12,14-16]. The C2IsH represents the vertical dimension of the pars interarticularis between the superior articular facet and the vertebral artery groove, while the internal height corresponds to the vertical height of the C2 lateral mass adjacent to the vertebral artery groove [24-26]. Reduction in these dimensions reflects encroachment of the vertebral artery into the pedicle region, leaving insufficient bone stock for safe pedicle screw insertion and significantly narrowing the operative corridor [6-10,26,27].

Additional radiographic parameters have been proposed to further characterize high-risk anatomy. Several studies incorporate C2 pedicle width (C2PW) ≤4 mm as an additional criterion, identifying patients with narrow pedicles who may be at increased risk for cortical breach or vascular injury during instrumentation [16-18]. Axial CT measurements also allow evaluation of pedicle morphology and laminar dimensions, including C2 lamina width (C2LW), which is particularly relevant when considering alternative fixation techniques such as laminar screw placement in cases of unfavorable pedicle anatomy [16-18]. These measurements are typically obtained using multiplanar CT or CTA reconstructions, which provide accurate visualization of both vascular and osseous structures and improve preoperative risk assessment [19-21].

More recently, classification systems have been proposed to further subcategorize HRVA based on the specific anatomical parameters involved [18,22]. A commonly used system distinguishes three primary HRVA types according to which measurements are reduced [18,24,25]. Type 1 (isthmic) HRVA is defined by reduced C2IsH (≤5 mm) with preserved C2InH and represents the most common pattern, accounting for approximately 78.2% of cases [22,23]. Type 2 (internal) HRVA is characterized by reduced C2InH (≤2 mm) with preserved C2IsH and occurs in approximately 8.8% of cases [24,25]. Type 3 (isthmo-internal) HRVA involves simultaneous reduction of both C2IsH and C2InH and accounts for approximately 12.9% of cases [24,25]. Expanded classification systems have described rare variants, including Type 4 (isthmio-absent) and Type 5 (internal-absent), in which one of the measurements approaches zero; however, these configurations are exceedingly uncommon, occurring in fewer than 0.2% of patients [19,24,25]. Interestingly, an alternative Yang Classification categorizes vertebral artery variations more broadly at the craniovertebral junction, with HRVA designated as Type B2 (31.4% prevalence) within a comprehensive 10-subtype system that includes both osseous and non-osseous vertebral artery anomalies [26]. 

These radiographic definitions and classification systems underscore the importance of careful preoperative imaging in patients undergoing C1-C2 instrumentation [27,28]. Identification of HRVA enables surgeons to modify surgical strategy, including selection of alternative fixation techniques such as pars screws, laminar screws, or navigation-assisted instrumentation, in order to reduce the risk of VAI [27,28]. As CT-based anatomical and radiologic studies continue to evolve, improved standardization of HRVA definitions and integration with advanced imaging and navigation technologies may further enhance surgical planning and risk stratification for atlantoaxial procedures [27,28].

Prevalence of HRVA

The reported prevalence of HRVA varies considerably across studies, largely due to differences in imaging modalities, measurement criteria, and patient populations [14-16,21,24,29,30,31-33]. Table 1 summarizes prevalence across representative radiologic studies. Using commonly accepted radiographic thresholds, including reduced C2IsH (≤5 mm) and/or C2InH (≤2 mm), most CT-based investigations report HRVA prevalence rates ranging from approximately 10% to 25% in general populations [14-16,21,24,29,30,31-33]. The largest available meta-analysis, incorporating 20 studies and 3,126 subjects, reported a pooled prevalence of 25.3% (95% CI, 19.6%-31.5%) for at least one HRVA, highlighting that this anatomical variant is relatively common rather than rare [29].

**Table 1:** Prevalence of HRVA Across Selected Imaging Studies. Reported prevalence of HRVA varies widely depending on population characteristics and imaging criteria. Studies based on general populations typically report prevalence rates between approximately 10% and 25%, whereas higher rates may be observed in selected patient populations, including those with congenital craniovertebral junction anomalies or cervical spine pathology. HRVA: High-Riding Vertebral Artery

Study	Population	Sample Size	HRVA Prevalence
Klepinowski et al. (2020) [29]	Central European (meta-analysis)	3126	25.3%
Klepinowski et al. (2021) [30]	Polish population	388	25.3%
Vaněk et al. (2017) [21]	Czech population	511	24.1%
Wakao et al. (2014) [32]	Japanese population	387	10.1%
Du et al. (2025) [24]	Chinese population	1804	29.6%
Shimizu et al. (2021) [34]	Japanese patients with cervical spine disorders	215	21.4%
Yamazaki et al. (2012) [33]	Japanese patients with congenital skeletal anomaly patients at the craniovertebral junction	100	51.9%

Large-scale imaging studies further demonstrate substantial variability across geographic and demographic groups. In Central European populations, prevalence rates are consistently reported around 24%-25%, including 25.3% in Polish cohorts and 24.1% in Czech populations [21,30]. In contrast, some Asian populations demonstrate lower prevalence rates, with one Japanese CT-based study reporting 10.1% [32]. However, other Asian cohorts have shown higher rates; for example, a large Chinese study reported a prevalence of approximately 29.6%, with corresponding reductions in mean C2IsH and C2InH compared with Western populations [24]. These findings suggest that anatomical differences in pedicle morphology and vertebral artery position may vary by ethnicity and contribute to differences in surgical risk profiles, which should be taken into consideration during surgical planning [21,24,30,32].

HRVA prevalence is also significantly influenced by underlying pathology. In patients with congenital craniovertebral junction anomalies, prevalence rates may be markedly elevated [33]. One study reported HRVA in 51.9% of patients with atlantoaxial subluxation and congenital anomalies [33]. Similarly, in patients with cervical spine disorders, HRVA prevalence has been reported at approximately 21.4%, with a significant association between HRVA and degenerative changes such as C1-C2 facet osteoarthritis [34]. These findings underscore the importance of considering HRVA not only as an anatomical variant but also as a feature that may be associated with specific pathological conditions [33,34].

Patient-specific factors, including age and sex, have also been associated with HRVA [4,25,29,35-37]. Advanced age and female sex have been identified as independent predictors of HRVA presence in several studies [4,25,29,35,36]. Additionally, rheumatoid arthritis has been shown to increase the likelihood of HRVA, potentially due to chronic inflammatory changes affecting the craniovertebral junction and adjacent osseous structures [37]. In terms of laterality, HRVA is more commonly unilateral, occurring in approximately 70% of cases, while bilateral HRVA is observed in approximately 30% of patients, with no consistent left-right predominance [4,29].

Overall, the variability in reported prevalence likely reflects differences in imaging resolution, measurement methodology, and population characteristics. Despite this heterogeneity, the available literature consistently demonstrates that HRVA is encountered with sufficient frequency to warrant routine evaluation during preoperative planning [24,29,35,36]. Given its potential to significantly alter surgical risk, systematic risk assessment, with an understanding of patient-level risk factors, including the presence of specific cervical degenerative pathologies and potentially ethnic origin, can be beneficial before C1-C2 instrumentation [24,29,35,36].

Surgical implications

The presence of an HRVA has significant implications for surgical planning during cervical spine instrumentation, particularly at the C1-C2 level [4-6,15,16,31]. The vertebral artery courses through the transverse foramina and vertebral artery groove of C2, placing it in proximity to the pedicle and pars interarticularis, which are structures frequently used for screw fixation [4-6,15,16,31]. When HRVA is present, the available osseous corridor for safe screw placement is markedly reduced, substantially increasing the risk of VAI during instrumentation [15,16,26-29]. This risk is particularly relevant during the placement of C2 pedicle screws, which are widely utilized due to their superior biomechanical stability and fixation strength [26-29]. However, multiple anatomical and radiologic studies have demonstrated that reduced pedicle height and medialization of the vertebral artery significantly compromise the safety of pedicle screw placement in HRVA patients [5,6,13,15].

From a surgical perspective, HRVA alters both the feasibility and safety margin of standard instrumentation techniques. Even minor deviations in screw trajectory may result in cortical breach or vascular injury due to the reduced tolerance within the pedicle corridor [31,38-42]. Freehand techniques, in particular, may be less reliable in this setting, as they depend heavily on consistent anatomical landmarks that are distorted in HRVA [42]. As a result, surgeons must carefully evaluate preoperative imaging to determine whether pedicle screw placement is safe or whether alternative strategies are warranted [42].

HRVA significantly increases the risk of VAI during C2 instrumentation, with overall arterial injury rates of 1.35% for C1-C2 posterior fixation compared to 0.08% for all cervical spine surgeries [24,43]. The VAI rate per screw placed is approximately 1%, with a 2% per-patient rate during C1-C2 posterior fusion [44]. The most common injury mechanisms include screw insertion (31% of cases), high-speed drilling (23% of cases), and, less commonly, rod compression of the vertebral artery [37,45]. C1 lateral mass screws and C2 pedicle screws account for the majority of screw-related injuries [43].

Management of HRVA injury

Management of VAI in the setting of HRVA depends on the location of injury, severity of bleeding, and vertebral artery dominance [8,46-48]. Although VAI is relatively uncommon, it represents one of the most serious complications of cervical spine instrumentation and requires prompt recognition and intervention [8,46-48]. The presence of HRVA increases the risk of injury due to reduced pedicle dimensions and altered vascular anatomy, making both prevention and management critical components of surgical planning [8,47,48].

Immediate control of bleeding is the primary objective when VAI occurs [8,37,46-48]. Tamponade is typically the first-line intervention and can often achieve hemostasis, particularly when the injury occurs within a screw tract or bony canal [37,47,48]. In these cases, screw insertion or packing may compress the injured vessel against surrounding bone and effectively control bleeding [47,48]. In contrast, injuries occurring in open surgical fields may result in uncontrolled hemorrhage that cannot be managed with tamponade alone [37,46,47]. In such cases, prompt endovascular consultation is required, as embolization or vessel occlusion may be necessary to achieve definitive hemostasis [37,46,47]. Hemostatic agents such as gelatin sponge, oxidized cellulose, and fibrin sealants may provide temporary control but should not be considered definitive management in unstable cases [37,46,47].

Before definitive management, assessment of vertebral artery dominance and collateral circulation is essential [8,48]. Failure to evaluate vascular dominance before vessel occlusion significantly increases the risk of ischemic complications, with one study reporting neurologic sequelae in up to 41% of cases when vascular status was unknown [8]. Determining whether the injured artery is dominant, co-dominant, or non-dominant is therefore critical in guiding management decisions [8].

Definitive management strategies include direct surgical repair and endovascular intervention [8,48]. When technically feasible, direct repair of the vertebral artery has demonstrated favorable outcomes and may preserve vessel patency [8]. However, due to the anatomical complexity of the region, endovascular techniques are increasingly utilized [48].

Endovascular treatment includes two primary approaches: vessel preservation and vessel sacrifice [49,50]. Stent placement allows reconstruction of the injured artery while maintaining flow and has demonstrated high success rates, with angiographic success approaching 99% and low rates of stroke and mortality [46,49,50]. Alternatively, embolization or vessel occlusion may be performed in cases of uncontrollable bleeding, provided that adequate collateral circulation is confirmed [37]. In one series, endovascular repair was successfully performed in 13.3% of VAI cases without long-term neurologic deficits [46].

In cases where hemostasis is achieved without vessel sacrifice, antithrombotic therapy in the short term, involving either antiplatelet or anticoagulation therapy, may be considered to reduce the risk of thromboembolic complications [51,52]. Current recommendations support the use of anticoagulation therapy for at least three months for acute ischemic syndromes with angiographic evidence of thrombus [51,52].

Delayed complications remain a significant concern. Pseudoaneurysm formation has been reported in up to 48% of cases managed with tamponade alone, and delayed hemorrhage or arteriovenous fistula may occur even after initially successful management [8,53]. Therefore, follow-up imaging with CTA or MR angiography is recommended to monitor for vascular complications.

VAI cases during instrumentation may remain asymptomatic, but serious complications can occur in a significant minority of patients [39,41,42,46]. One systematic review found that VAI during atlantoaxial instrumentation had the potential to result in catastrophic complications [46]. It was found that ipsilateral stroke occurs in 10% of cases, arteriovenous fistula in 8.3% of cases, non-persistent neurologic deficit in 6.7% of cases, pseudoaneurysm in 3.3% of cases, permanent neurologic deficit in 1.7% of cases, with mortality occurring in 6.7% of cases [46]. Interestingly, the occurrence of stroke showed no significant correlation with injury severity, which can make predicting clinical outcomes very challenging [43]. Moreover, these complications underscore the importance of recognizing HRVA as a high-risk anatomical variant.

Given the potentially catastrophic consequences of VAI, prevention remains paramount, particularly in patients with HRVA. Preoperative CTA is essential to identify vascular anomalies and guide surgical planning [19-21]. Intraoperative strategies such as navigation systems, fluoroscopic guidance, and Doppler ultrasonography can further reduce the risk of vascular injury [26-29,39-42].

Importantly, the presence of HRVA not only affects intraoperative decision-making but also influences overall surgical strategy [13,42]. In many cases, the identification of HRVA precludes safe use of traditional pedicle screw fixation and necessitates alternative approaches to maintain stability while minimizing vascular risk [5,6,13,42]. Furthermore, HRVA may limit bilateral pedicle screw placement, requiring asymmetric or hybrid constructs depending on laterality and severity of anatomical compromise [42,44]. As such, HRVA should be considered a key determinant in preoperative planning, influencing both fixation strategy and operative technique [42,46].

Alternative surgical fixation techniques 

One retrospective study demonstrated that the presence of HRVA in patients with basilar invagination significantly increases the risk of pedicle screw misplacement when using freehand techniques, highlighting the limitations of conventional approaches in this population [42]. The same study emphasized that trajectory modifications are critical in HRVA cases; compared with normal anatomy, the optimal entry point should be shifted superiorly, with increased cephalad and medial angulation to accommodate altered vascular anatomy [38,42,54,55]. Despite these adjustments, safe pedicle screw placement may still be limited in many patients, necessitating alternative fixation strategies [38,42]. Accordingly, several alternative fixation techniques have been developed to maintain biomechanical stability while avoiding high-risk screw trajectories [38,42,54,55].

Among these, the C2 pars screw is one of the most commonly utilized alternatives [42,56]. By following a shorter and more medial trajectory, pars screws avoid the vertebral artery groove in many cases and provide a technically straightforward solution [42,56]. Although they offer slightly reduced biomechanical strength compared with pedicle screws, they remain a reliable and widely accepted option in HRVA patients, particularly when pedicle anatomy is unfavorable [42,56].

C2 laminar (translaminar) screws represent another widely adopted technique [57]. This approach involves placement of bilateral crossing screws through the C2 lamina, thereby completely avoiding the pedicle and vertebral artery [57,58]. Biomechanical studies have demonstrated comparable fixation strength to pedicle screws in many clinical scenarios, making this an attractive option in high-risk anatomy [57,58]. However, clinical outcomes may vary depending on patient pathology. For example, one study reported higher rates of instability and reoperation in patients with atlantoaxial dislocation treated with translaminar fixation [59], suggesting that patient selection remains critical.

Hybrid constructs, combining C1 lateral mass screws with C2 pars or laminar fixation, are also commonly employed to balance stability and safety [38,45,59,60]. These constructs are particularly useful in cases of unilateral HRVA or asymmetric anatomy, allowing tailored fixation strategies that avoid high-risk regions [45,59,60]. However, even with modified constructs, the risk of vertebral artery groove violation remains significant in patients with severely compromised pedicle anatomy. Yeom et al. demonstrated that in patients with narrow pedicles, rates of vertebral artery groove violation remained high regardless of fixation technique, with no significant difference between modified constructs and traditional pedicle screw placement [61].

Navigation-assisted instrumentation has also been shown to improve screw placement accuracy in anatomically constrained corridors such as HRVA, reducing the risk of VAI compared with freehand techniques [23,62]. Several less conventional techniques have also been described. The C2 medial window screw, which involves intentional medial pedicle breach, has been proposed as a method to avoid VAI [63]. Similarly, the C2 partial transpedicular screw (C2PTS) technique allows three-column fixation and may serve as an effective alternative when combined with careful inferior mobilization of the vertebral artery [64]. Other approaches include insertion of a C2 subfacetal screw into the vertebral body following vertebral artery mobilization to preserve vascular integrity [65]. Additionally, Neo et al. described the use of an aiming device to facilitate safe screw trajectory during atlantoaxial fixation and reduce the risk of vascular injury [66,67]. More recently, alternative entry techniques using a high-speed burr to define the pedicle entry point have also been reported as safe and feasible in patients with HRVA [55].

Finally, vertebral artery mobilization has been described as an adjunctive technique in selected high-risk cases [68]. Careful exposure and controlled mobilization of the vertebral artery can expand the operative corridor and facilitate safe screw placement when conventional trajectories are not feasible [68-70]. However, this approach requires advanced surgical expertise and therefore should be reserved for carefully selected cases [68-70]. 

These strategies are summarized in Table 2, and the selection of the appropriate technique depends on patient-specific anatomy, surgeon experience, and the presence of vascular anomalies.

**Table 2:** Alternative Fixation Techniques for C2 Instrumentation in the Setting of HRVA. Various surgical techniques have been described to mitigate the risk of vertebral artery injury in patients with HRVA. Selection of the optimal approach depends on patient-specific anatomy, surgeon experience, and available intraoperative resources. While pedicle screws provide superior biomechanical stability, alternative techniques, such as pars screws, laminar screws, and modified constructs, are commonly employed to avoid high-risk vascular anatomy. HRVA: High-Riding Vertebral Artery

Fixation Technique	Description	Advantages	Limitations	Key References
C2 Pedicle Screw (modified trajectory)	Pedicle screw placement with adjusted entry point and angulation based on HRVA anatomy	Maintains the strongest biomechanical fixation; familiar technique	Narrow safe corridor; high risk of vertebral artery injury without navigation or modification	Shao et al. [56]; Yeom et al. [61]; Arslan et al. [23]
C2 Pars Screw	Screw inserted through the pars interarticularis instead of pedicle	Avoids vertebral artery groove; technically straightforward	Shorter screw length; slightly reduced biomechanical strength	Shao et al. [56]
C2 Laminar (Translaminar) Screw	Bilateral crossing screws placed through the C2 lamina	Completely avoids pedicle and vertebral artery; safe in HRVA	Limited in patients with thin laminae; may have inferior outcomes in certain pathologies	Doward et al. [57]; Meyer et al. [58]
Navigation-Assisted Pedicle Screw	Pedicle screw placement guided by intraoperative navigation or imaging	Improves accuracy; may allow safe pedicle fixation in borderline anatomy	Requires specialized equipment and experience	Arslan et al. [23]; Malikov et al. [62]
Modified C1-C2 Constructs	C1 lateral mass screws combined with C2 pars or laminar fixation	Provides stable fixation while avoiding the pedicle	More complex construct; may require greater exposure	Harms et al. [60]
C2 Partial Transpedicular/Alternative Pedicle Techniques	Modified pedicle-based techniques (e.g., partial transpedicular screws) to preserve the safe corridor	Allows pedicle-based fixation while minimizing vertebral artery risk	Technically demanding; limited data	Guo et al. [63]; related studies [55,64,65-67]
Vertebral Artery Mobilization (adjunct technique)	Surgical exposure and mobilization of the vertebral artery to expand the operative corridor	Enables pedicle or pars screw placement in otherwise unsafe anatomy	Technically demanding; risk of vascular injury if improperly performed	Goel et al. [68]; Liu et al. [69]

Role of preoperative imaging

Preoperative imaging plays a crucial role in identifying HRVA and guiding safe surgical planning [19-22]. High-resolution CT and CTA are widely considered the most reliable modalities for evaluating vertebral artery anatomy and pedicle morphology before cervical instrumentation [19-22,26,32,33]. Multiplanar CT reconstructions allow precise measurement of C2 pedicle dimensions, including isthmus height, internal height, and pedicle width, which are essential for identifying HRVA and determining the feasibility of pedicle screw placement [19-22,26,32,33]. In particular, CT-based morphometric analysis enables surgeons to quantify the available osseous corridor and assess whether standard screw trajectories can be safely achieved [19,26,32,33].

CTA further enhances preoperative assessment by providing detailed visualization of the vertebral artery course relative to surrounding osseous structures and allows identification of vascular anomalies such as medialization, duplication, or aberrant entry levels [71,72]. Importantly, CTA also provides information regarding vertebral artery dominance, which is critical for surgical decision-making and risk stratification in cases where vascular compromise is possible [71,72]. Compared with nonenhanced CT or MRI, CTA offers superior sensitivity for detecting vertebral artery anomalies, while maintaining high specificity, making it the imaging modality of choice in high-risk patients [19-21].

The combination of CT and CTA allows simultaneous evaluation of both osseous and vascular anatomy, facilitating accurate preoperative planning and reducing the likelihood of intraoperative complications [19,52]. Several studies have emphasized the importance of routine preoperative CT and CTA assessment of the C2 pedicle and vertebral artery groove when planning atlantoaxial fixation [23,42]. This is particularly important in patients with suspected HRVA or other anatomical variations, where reliance on standard anatomical landmarks alone may be insufficient [23,42].

In addition, emerging imaging technologies such as three-dimensional (3D) reconstruction and virtual surgical planning have further improved preoperative evaluation [68,73]. These techniques allow surgeons to simulate screw trajectories and assess risk in a patient-specific manner, potentially improving accuracy and reducing complication rates [68,73]. As such, comprehensive preoperative imaging should be considered essential in all patients undergoing C1-C2 instrumentation, particularly when HRVA is suspected [23,42,68,73].

Risk mitigation strategies 

In addition to careful preoperative imaging, several intraoperative strategies have been developed to mitigate the risk of VAI in patients with HRVA [23,62,74]. One of the most important approaches is the use of image-guided navigation systems, which provide real-time visualization of screw trajectories relative to patient-specific anatomy [23,62,74]. Navigation-assisted instrumentation has been shown to significantly improve screw placement accuracy in anatomically constrained corridors such as HRVA, reducing rates of pedicle breach and vascular injury compared with freehand techniques [23,62,74]. In particular, intraoperative CT-based navigation systems, such as O-arm imaging, allow precise localization of the vertebral artery and pedicle boundaries, with reported reductions in VAI rates compared with conventional fluoroscopy alone [74].

Fluoroscopic guidance remains a commonly utilized method for confirming screw trajectory during instrumentation, particularly in settings where advanced navigation systems are not available [74-76]. However, fluoroscopy provides limited visualization of vascular structures and should be supplemented with additional techniques in high-risk cases [74-76]. Intraoperative Doppler ultrasonography represents another valuable tool, allowing real-time identification of the vertebral artery and confirmation of its location before drilling or screw placement [75,76]. Studies have demonstrated that the use of Doppler guidance can reduce the risk of vascular injury by improving intraoperative awareness of arterial position [75,76].

Meticulous surgical technique is equally critical in minimizing risk [23,54-63,65-71]. Careful exposure, avoidance of excessive drilling, and adherence to planned trajectories are essential, particularly in the anatomically complex atlantoaxial region [23,54-63,65-71]. In selected cases, controlled mobilization of the vertebral artery may be performed to expand the operative corridor and facilitate safe screw placement [68-70]. While this technique can be effective, it requires advanced surgical expertise and, therefore, should be reserved for carefully selected patients [68-70].

Importantly, risk mitigation in HRVA extends beyond intraoperative technique to include strategic modification of instrumentation [23,54-63,65-67]. When HRVA is identified preoperatively, surgeons may elect to avoid pedicle screw placement altogether and instead utilize alternative fixation techniques such as pars screws, laminar screws, or hybrid constructs, as discussed previously [23,54-63,65-67]. Adjustment of screw trajectory, including more medial or cephalad angulation, may also reduce vascular risk in borderline cases [23,54,61]. However, even with these modifications, the margin for error remains limited, emphasizing the importance of individualized surgical planning [23,61].

Finally, prompt recognition of intraoperative VAI remains a critical component of risk mitigation [8]. Vascular injury typically presents with sudden, profuse bleeding, most commonly during drilling or instrumentation [8,37,47,49,77]. Delayed presentations may include pseudoaneurysm, arteriovenous fistula, or compressive symptoms, underscoring the importance of early recognition and appropriate vascular evaluation [39,41,42,46,77]. Early identification allows for immediate control and appropriate escalation of management, including tamponade, direct repair, or endovascular intervention [39,41,42,46,77]. Table 3 summarizes preoperative imaging findings and corresponding risk mitigation strategies in patients with HRVA.

**Table 3:** Preoperative Imaging Findings and Risk Mitigation Strategies in High-Riding Vertebral Artery. Careful evaluation of C2 pedicle morphology and vertebral artery position on CT or CT angiography, is essential for safe surgical planning. Specific anatomical findings, including reduced pedicle height, narrow pedicle width, and medialization of the vertebral artery, are associated with increased risk of vascular injury and should prompt consideration of alternative fixation techniques or image-guided instrumentation. CT: Computed Tomography

Imaging Finding	Surgical Risk	Recommended Surgical Consideration
Reduced C2 pedicle height (C2IsH ≤5 mm)	Narrow safe corridor; increased risk of vertebral artery injury during pedicle screw placement	Avoid C2 pedicle screw; consider pars or laminar screw fixation
Reduced C2 internal height (C2InH ≤2 mm)	Superior displacement of the vertebral artery encroaching on the pedicle trajectory	Avoid pedicle screw; use alternative fixation techniques
Narrow C2 pedicle width (≤4 mm)	Limited screw diameter and trajectory; higher risk of cortical breach	Consider a pars screw, laminar screw, or navigation-assisted placement
Medialized vertebral artery	Increased likelihood of vertebral artery violation even with standard trajectories	Utilize navigation-guided instrumentation or alternative fixation
Severely compromised pedicle anatomy	Inadequate bone stock for safe pedicle screw placement	Prefer laminar screw fixation or modified C1-C2 constructs
Bilateral HRVA or complex vascular anatomy	Limited safe fixation options bilaterally	Consider laminar screws, hybrid constructs, or staged fixation strategies
High-risk anatomy on CT/CTA (combined HRVA + narrow pedicle)	Significantly elevated risk of intraoperative vascular injury	Preoperative planning with CTA; avoid freehand technique; consider navigation
Intraoperative concern for vascular proximity	Risk of unrecognized vertebral artery injury	Use fluoroscopy, navigation, or Doppler; consider vertebral artery mobilization in select cases

Limitations of current literature 

Despite increasing recognition of HRVA as a clinically significant anatomical variant, the current literature remains limited in several important ways. The majority of available studies are retrospective radiologic or cadaveric investigations that primarily focus on anatomical characterization of the C2 pedicle and vertebral artery course, rather than clinical outcomes or surgical decision-making [4,12,13,29]. As a result, although these studies have been instrumental in defining HRVA and quantifying its prevalence, their ability to directly inform operative risk and complication rates is limited [4,12,13,29].

Furthermore, there is a relative lack of prospective clinical studies evaluating the impact of HRVA on surgical outcomes, including VAI, screw accuracy, and long-term construct stability [39,78]. Much of the current understanding of injury risk is extrapolated from broader cervical spine literature rather than HRVA-specific cohorts, making it difficult to determine the true magnitude of risk attributable to this anatomical variant alone [4,6,12,29,42,59]. In addition, many surgical studies are single-center and involve relatively small sample sizes, limiting generalizability across diverse patient populations and surgical techniques [33,34].

Another major limitation is the lack of standardized radiographic definitions of HRVA. Although commonly used criteria include C2IsH ≤5 mm and/or C2InH ≤2 mm, variations in measurement techniques, imaging planes, and threshold values exist across studies [12,15,18-25]. This heterogeneity complicates comparisons of prevalence, risk stratification, and surgical decision-making between studies. Differences in imaging modality (CT vs CTA), slice thickness, and reconstruction methods may further contribute to variability in reported measurements and classification of HRVA [19-22,42].

Population heterogeneity also represents an important limitation [21,24,29,30,32-34]. Many studies are derived from specific geographic or ethnic cohorts, and growing evidence suggests that HRVA prevalence and pedicle morphology may vary across populations [21,23,29,30,32-34]. Additionally, patient-specific factors such as age, sex, degenerative disease, rheumatoid arthritis, and congenital craniovertebral junction anomalies may influence both the presence of HRVA and its surgical implications [21,24,29,30,32-34]. However, these associations are not consistently accounted for across studies [21,24,29,30,32-34].

Importantly, most studies emphasize anatomical feasibility rather than comparative effectiveness of different fixation strategies. There is limited high-quality evidence comparing outcomes between pedicle screws, pars screws, laminar screws, and hybrid constructs specifically in HRVA patients [23,54-67]. Similarly, the role of adjunct techniques such as navigation, Doppler ultrasonography, and vertebral artery mobilization has not been systematically evaluated in larger cohorts [68-70,75,76]. As a result, surgical decision-making remains largely based on surgeon preference and institutional experience rather than robust comparative data [68-70,75,76].

Finally, reporting of VAI remains inconsistent [39,41,42,46,77,78]. Many studies rely on intraoperative recognition of VAI, while delayed complications such as pseudoaneurysm or arteriovenous fistula may be underreported due to a lack of standardized follow-up imaging protocols [39,41,42,46,77,78]. This likely leads to underestimation of true complication rates and limits the ability to fully assess the clinical significance of HRVA [39,41,42,46,77,78].

Future directions 

Future research should focus on improving both the identification and surgical management of HRVA through advances in imaging, classification systems, and operative planning. High-resolution CTA remains the main imaging modality for preoperative evaluation, but emerging technologies such as 3D reconstruction and virtual surgical simulation offer the potential for more precise, patient-specific assessment of vertebral artery anatomy and pedicle morphology [73]. These tools may allow surgeons to preoperatively model screw trajectories and identify high-risk anatomy with greater accuracy [73].

Standardization of radiographic definitions and classification systems for HRVA represents a critical area for future work [24,25]. The development of universally accepted measurement criteria and classification schemes would improve consistency across studies and facilitate more reliable comparisons of prevalence, risk, and outcomes [24,25]. Integration of both osseous and vascular parameters into unified classification systems may further enhance clinical applicability [24,25].

Prospective multicenter studies are also needed to better evaluate HRVA outcomes, including screw accuracy with alternative fixation methods, VAI rates, neurologic complications, and long-term construct stability across different fixation techniques [30,42,79,80]. Comparative effectiveness research examining pedicle screws versus alternative techniques (e.g., pars, laminar, and hybrid constructs) in HRVA patients would be particularly valuable in guiding evidence-based surgical decision-making [30,42,79,80].

Advances in intraoperative technology also represent an important area of future investigation. Navigation-assisted and robotic-guided instrumentation have demonstrated improved accuracy in screw placement and may play an increasingly important role in managing complex anatomy such as HRVA [23,74,79]. Further studies are needed to evaluate whether these technologies translate into reduced complication rates and improved clinical outcomes.

In addition, the application of machine learning and artificial intelligence to imaging-based risk stratification is an emerging and promising field [81]. Recent work by Ye et al. (2025) demonstrated that models incorporating CT/CTA-derived anatomical parameters can achieve strong predictive performance for VAI risk, supporting the potential for automated risk assessment tools to aid surgical planning [81]. Future studies should aim to validate these models in larger, multicenter cohorts and integrate them into clinical decision-making.

Finally, the development of standardized management algorithms for HRVA, including imaging protocols, surgical decision-making pathways, and intraoperative risk mitigation strategies, may help improve consistency in care and reduce complication rates for patients with HRVA undergoing cervical surgery [48,82]. As imaging, navigation, and predictive modeling technologies continue to evolve, a more comprehensive and individualized approach to HRVA management will ultimately enhance surgical safety and patient outcomes.

## Conclusions

HRVA represents an important anatomical variant of the vertebral artery with significant implications for cervical spine instrumentation, particularly at the level of C1-C2, where the safe osseous corridor for pedicle screw placement is inherently limited. Recognition of HRVA is critical, as failure to identify this variant may result in catastrophic vascular injury with potentially severe neurologic consequences. Moreover, thorough preoperative evaluation with CT and CTA is essential to delineate vertebral artery anatomy and guide surgical planning accurately. In patients with HRVA, modification of instrumentation strategies, including the use of alternative fixation techniques, navigation-assisted approaches, or hybrid constructs, may be required to optimize safety while maintaining adequate biomechanical stability. Ultimately, an individualized and anatomy-driven approach to surgical decision-making is necessary to minimize complications and improve surgical outcomes in patients with HRVA.
